# Traditional Coping Strategies and Disaster Response: Examples from the South Pacific Region

**DOI:** 10.1155/2013/264503

**Published:** 2013-12-23

**Authors:** Stephanie M. Fletcher, Jodi Thiessen, Anna Gero, Michele Rumsey, Natasha Kuruppu, Juliet Willetts

**Affiliations:** ^1^World Health Organization Collaborating Centre for Nursing, Midwifery and Health Development, University of Technology, Sydney, P.O. Box 123, Broadway, NSW 2007, Australia; ^2^Institute for Sustainable Futures, University of Technology, Sydney, P.O. Box 123, Broadway, NSW 2007, Australia

## Abstract

The Pacific Islands are vulnerable to climate change and increased risk of disasters not only because of their isolated and often low lying geographical setting but because of their economic status which renders them reliant on donor support. In a qualitative study exploring the adaptive capacity of Pacific Island Countries (PICs) across four countries, Cook Islands, Fiji, Samoa, and Vanuatu, it was clear that traditional coping strategies are consistently being applied as part of response to disasters and climate changes. This paper describes five common strategies employed in PICs as understood through this research: recognition of traditional methods; faith and religious beliefs; traditional governance and leadership; family and community involvement; and agriculture and food security. While this study does not trial the efficacy of these methods, it provides an indication of what methods are being used and therefore a starting point for further research into which of these traditional strategies are beneficial. These findings also provide important impetus for Pacific Island governments to recognise traditional approaches in their disaster preparedness and response processes.

## 1. Introduction

There exists evidence that global warming is contributing to increased numbers of observed extreme weather events, such as heat waves, extremes in precipitation, and the potential for increased number of severe storms [1]. For the Pacific, projected frequency of tropical cyclones may follow global trends of less frequent tropical cyclones and a likely increase in the relative proportion of severe storms by the end of the 21st century [2]. Coping with climate and weather extremes is a natural part of Pacific Islander's way of life. For instance, the 1997-98 El Nino events were blamed for widespread drought and famine in some Pacific islands, including major agricultural losses in Fiji, and widespread famine in parts of Papua New Guinea [3, 4]. Tropical cyclones, increased rainfall, flooding, and cyclone induced storm surges have resulted in extensive infrastructural and financial losses; particularly in Samoa, Cook Islands, and Fiji [2, 5–7]. It is anticipated that with projected climate changes, patterns of extreme weather including tropical cyclones may change, with an increased proportion of severe storms [4]. The extensive coastal boundaries, the numerous islands and atolls, long coastlines, and the density of population in coastal areas of South Pacific countries have made tsunamis, floods, and tidal abnormalities the most destructive problems in the region [8, 9].

In the context of this paper, “climate change” refers to any change in climate over time, whether due to natural variability or as a result of human activity [10]. A UNICEF report in 2010 indicates that climate change impacts in the Asia and the Pacific region will result in mortality and injury from extreme weather events; increase in risk of water scarcity; changes in the transmission, incidence, and distribution of water-, food-, and vector-borne diseases; migration and declining livelihoods; and heightened risk of food insecurity and child malnutrition [11]. Moreover, in the Pacific Island Countries (PICs) context, there are several ‘‘nonclimate” related factors and processes which affect the ability of communities to cope with climate stresses. These include (but are not limited to) rapid population growth, land ownership and kinship issues, intrusion of western culture into traditional governance, the erosion of traditional knowledge impacting agricultural practices, natural resource degradation, and increasing reliance on imports [12, 13]. Additionally, the small population size and economic vulnerability exacerbated by frequent disasters have resulted in the need for humanitarian assistance and development aid to manage the effects of disasters and climate change across the region [14, 15]. Both Australian government, local, regional, and international nongovernmental organizations (NGOs) and multilateral organizations including United Nations agencies are key development partners and provide rapid humanitarian assistance in PICs [15–19]. Several of these organizations also work to ensure the disaster response process transitions to postdisaster development programs to enhance overall in-country capacity [6, 20–22]. Disaster preparedness, risk reduction, and climate change adaptation are some of the thematic areas of development assistance in PICs [6, 21–24].

Historically, however, Pacific populations have applied their own traditional and local knowledge to respond to environmental challenges and changes [25]. These local and traditional knowledge and skills are increasingly being recognised as vital resources for adaptation [26]. Understanding local strategies used to prepare for, respond to, and recover from climate related disasters will help to define the Pacific Islands capacity to adapt to disasters and climate related events [25, 27]. This approach has being widely recognised and adopted within the field of climate change adaptation across developing countries as a part of response [28–30]. Both international and local leaders across the Pacific recognise the importance of traditional knowledge and coping skills to combat the effects of a changing and variable climate [14, 23, 30–33]. The Pacific Islands Framework for Action on Climate Change (PIFACC) [23] recognises and promotes the understanding and application of traditional knowledge to inform adaptation measures. To this end there has been a call for more research to link historical and anecdotal evidence of local coping strategies and for the integration of traditional knowledge into information management systems [23, 31]. Efforts have been made to capitalise on existing knowledge of regional cultural approaches and experiences with climate variability through consultative and research approaches [31]. It is therefore important for national disaster organizations and donors to recognise and incorporate traditional knowledge and skills in relevant planning and programming.

The purpose of this paper is to explore traditional coping strategies that may enhance long-term adaptive capacity to a variable and changing climate by looking specifically at examples from Pacific Islanders in their response to impacts of climate change and associated disasters. It draws on findings from a larger study which examined the adaptive capacity of the disaster response systems in four PICs to meet immediate humanitarian needs following a disaster [27]. This paper details the findings from the larger study giving examples of traditional coping strategies being used to address climate change impacts, including disasters, in terms of recognition of traditional coping strategies; faith and religious beliefs; traditional governance and leadership; family and community involvement; and agriculture and food security. These findings are useful for donor organizations and nongovernmental organizations to inform design and implementation of culturally relevant disaster risk reduction and climate change adaptation programs, in PICs and similar small island developing states.

## 2. Methods

A qualitative research methodology was used including desktop review of the literature, policy analysis, and ninety interviews with high level professionals involved in disaster response from Australian and PIC government ministries, local and regional NGOs, and international humanitarian and donor organizations. The research process was guided by a Project Reference Group (PRG) comprising key experts from Australia and the Pacific as a form of structured stakeholder oversight and engagement in the research. Furthermore, a Conceptual Framework [27] was developed to provide the scope for this study. This framework described a cycle of adaptive learning within which the adaptive capacity of the “disaster response system” (DRS) is influenced by a range of key determinants [34]. Specific determinants of adaptive capacity were used to assess the DRS and drew upon literature spanning Earth System Governance [35], climate change adaptation [34], health resources [36–38], resilience in institutions [39], and practice theory [40]. Key determinants of adaptive capacity were identified as being interorganizational including architecture; agency; adaptiveness [35, 41]; intraorganizational *objective* including leadership, management, and governance structures [42]; technical capacity, tools, methods, and approaches [43]; human resource for health governance and management systems [36, 37]; and finally intra-organizational *subjective* including risk perceptions [34]; self-efficacy beliefs [35, 39]; elements of social practice [40]; and traditional coping strategies [40, 44]. The subjective determinant of “traditional coping strategies” is the focus of this paper and therefore expands specifically on findings regarding this key determinant [27].

A semistructured interview guide informed by a conceptual framework was used to guide face-to-face and telephone interviews with all 90 participants; and probing conducted as needed. The interview style, developed with the guidance of the PRG, was designed to appreciate cultural differences including the need for clear questions and flexibility when interviewing people from various cultural backgrounds. Relevant organizations were identified with the assistance of the PRG, with additional organizations identified through a snow ball sampling technique (Atkinson and Flint, 2001). The interviews were conducted in both Australia and four Pacific Island Countries in May–July 2012 along with workshops with in-country stakeholders to test the preliminary analysis of interviews.

Four PICs were investigated in detail to provide more of a regional viewpoint: Cook Islands, Fiji, Samoa, and Vanuatu. These countries were selected with the aim to include a variety of geographical settings (including Melanesia and Polynesia); varied policy landscapes (regarding climate change, disaster, and health-related policies and plans); mix of countries with recent significant climate-driven disasters and those in the more distant past (relating to degrees of memory and levels of preparedness); and countries that experience tropical cyclones as an example of a rapid onset disaster. The scope of the study was limited to health related humanitarian needs and as such researchers were interested in obtaining a mix of countries including high and low HRH density. Development indicators (e.g., varied Human Development Index, see UNDP, 2011) were also used as a criterion for selection to include countries at different stages of development. A full account of the methodology can be found in [27].

## 3. Results and Discussions

Historically, the study of the traditional knowledge was the purview of anthropologists and sociologists. However, other disciplines have come to recognise the importance of the broader application and use of this knowledge before it disappears; and the value of the application of traditional methods to cope with environmental hazards is now being widely recognised [33, 45]. External influences from western colonisation, governance reforms, and development assistance have contributed to some loss of traditional and local knowledge and coping strategies [25]. Increasing commercialisation and urbanisation and the subsequent erosion of traditional social networks have also contributed to the loss of traditional knowledge and practices [13]. Within a generation traditional pass times centring on the extended family or local community have been replaced by individualised and home-based recreation [13]. Historically, livelihoods were constructed and maintained in a way that accounted for climate stress based on generations of experience of living with environmental uncertainty [12].

Members of the Pacific community recognise that with time, traditional knowledge is eroding due to a combination of factors such as migration, urbanization, and the passing away of elders [32]. It is essential therefore that beneficial traditional knowledge and cultural values are used and preserved in order to improve the ability of Pacific Islanders to build long-term resilience to natural disasters over time [32].

It became evident throughout the interviews conducted in PICs that traditional coping strategies are an important part of life and where some have been eroded, in particular with agriculture and food security, there is a current move back to integrating traditional methods, to improve disaster preparedness and response. The following section provides examples from the respondents as well as from the literature, outlining Pacific Islanders traditional coping strategies for disaster response and climate change adaptation.  Table 1 summarises findings around traditional knowledge and coping strategies from the Pacific Region. However, it is recognised that continuing cultural and demographic change across the region is likely to influence traditional disaster-related coping mechanisms, including those mentioned above.

## 4. Recognition of Traditional Coping Strategies

Disaster response stakeholders in four Pacific Island Countries indicated that traditional coping strategies were important to help communities cope with the effects of disasters and, increasingly, the impacts of climate change. The importance of recognising and applying traditional knowledge and coping strategies was widely recognised by interviewees. A NGO representative from Vanuatu summed it up succinctly.

“*We are adaptable and will find new ways to integrate science and traditional knowledge. There are traditional skills–food security and local capacity*.”

Government, donor and NGO communities across all countries agreed that there was “*a lot of capacity that can be tapped into the traditional cultures* …. *there is great potential here because of strong communities in place*.”

Unfortunately, traditional strategies were found to not always be recognised, relied on, and incorporated in national disaster response mechanisms. This may be due to the fact that “*the traditional coping methods documentation is not there, it's oral.” *According to one regional health respondent in Vanuatu,

“*There are strong partnerships between islands. This includes strong kinship ties. People develop good coping skills and foster relationships…. that are very important even during coordination of teams. We have local mechanisms in place that we (at the national level) seem to overlook. Legally, these traditional structures are not incorporated. It needs to become part of the local ways of responding.*”

Also a survey has indicated that the use of traditional medicine is not often acknowledged because of its association with “witchcraft” [46] (cited by WHO 2001). However, a health sector respondent indicated progress had been made with the successful publication of the traditional medicine book, Na Wai Vakaviti [47], by the Women's Association for Natural Medicinal Therapy, (Wainimate) Fiji. The respondent went on to say that “*we included the knowledge and the practice from those who have been practicing; they documented it and some those *[*traditional medicinal therapies*]* have been tested in the science lab in the University of South Pacific*.”

In this study, traditional practices were chosen for illustration based on their frequency of use or discussion by responders and based on first hand experiences gained by the researchers whilst working in the region. According to WHO, there is increasing recognition amongst PICs for the need to regulate the use of traditional medicines [48, 49], although for the most part this has not yet occurred. This is due to the fact that harmful and adverse reactions can result from the use of poor quality or inappropriately administered traditional medicines and practices [49, 50]. In recognition of the importance of the use of safe and effective traditional medicinal practices and products as an important form of health care for many people in the Western Pacific Region and to maximize the potential contribution of traditional medicine to primary health care, the WHO has developed the Regional Strategy for Traditional Medicine in the Western Pacific, as a guide to relevant stakeholders [49]. However, there is the need for more empirical evidence to determine the efficacy of traditional medicine practices and products, their use in conjunction with contemporary medicine, and the need for adequate information to guide consumers to make informed decisions [49].

In recognition of the strong traditional systems in the Pacific, the Climate Change and Development (CCD) Community, of the online Pacific Solution Exchange, recently held an online discussion that called for the incorporation of traditional knowledge into national planning in order to address climate change impacts and disaster risk reduction and to balance modern technology and science with such traditional practices in the PICs. According to a member of the CCD community, “*there is keen interest among both older and younger generations of islanders to record this traditional knowledge and to integrate it with scientific approaches*” [32].

Advancements have already been made in the preservation of *traditional knowledge.* For instance, the Climate Change and Development Community reported some examples which includes list of crops from the Secretariat of the Pacific Community's (SPC) climate ready collection and glossaries of traditional climate change terms in some Pacific countries, such as the iTaukei Glossary in Fiji. There was also a call for the inclusion of traditional knowledge in school curriculums, especially in remote areas with limited or no access to the internet. There was acknowledgement by several interviewees of the need to embrace traditional knowledge as an important and essential tool for disaster risk management and to combat climate change. This will require the combination of traditional knowledge with scientific knowledge, in order to promote the environmental, economic and social sustainability necessary when working with local communities who will be the primary beneficiaries of the products. To achieve this union, careful assessment and analysis are required to ensure that innate difficulties of blending traditional knowledge with scientific knowledge are addressed and that traditional value systems are upheld [32]. For instance, the Red Cross has introduced a toolkit for Pacific Red Cross societies and island communities to assess their vulnerability and capacity in times of natural disasters. Several techniques to raise community awareness were included in the toolkit, including [51] the following:

- recording of seasonal patterns over time,
- documenting social changes over time that impact lives and conditions, such as local hazards, construction material and design, vegetation, livestock, and environmental changes,
- mapping of risks, hazards, and disasters response resources,
- sharing of knowledge between communities,
- formal approval for traditional knowledge and disaster preparedness to be included in primary school curricula, planning, and social development programs,
- effective risk and hazard communication between communities and local authorities.

It must be noted, however, that, in the quest to blend science and traditional knowledge, care should be taken to ensure that practices that have a deleterious effect on populations are not reinforced. This is because not all traditional coping strategies are beneficial and some, in fact, can be harmful and infringe on human rights [52]. One such example was cited by a respondent in Samoa. After the tsunami in 2009, some first responders to the affected area prioritised the removal of the dead bodies before the injured, because it was a culturally acceptable way to pay respect to the dead. Prioritising the dead over the injured, thus delaying treatment, could result in deterioration of their health status. There is limited published information about the incidence and impact of some harmful traditional and cultural practices, and in many cases, these practices may not be seen as being harmful in their culture. Engaging with local communities to determine their attitudes to certain practices as well as to undertake further studies on the incidence, causes and consequences of harmful practices are recommended.

## 5. Faith and Religious Beliefs

Pacific Islands are well known for their strong religious ties and the involvement of religious groups in the development and leadership of communities. This study found that faith (predominantly Christianity) and involvement in religious communities were an important coping strategy across the case study countries. In fact, the Council of Churches or equivalent organization in each country was an important social and cultural institution and active in disasters response. Churches were found to play an important role in community life and often used as emergency centres or shelters in times of disaster. This church-based respondent described their community disaster evacuation plan:

“*Everyone is instructed that once they hear the warning they should come to the shelter (church building) with their emergency backpacks [which are already prepared with emergency supplies to last 48 hours] and walk up to the [designated] Primary School.*”

The role of the church is widely recognised across all levels of society. According to one NGO representative in Vanuatu, “*women's church groups are the strongest community group*.” The role of the church in posttsunami response in Samoa was often highlighted by respondents. This included the role of the Church as a key agent in the provision of counselling and psychosocial support to survivors and affected families and response personnel, bringing comfort to the people and attending to the spiritual needs of community and response personnel alike. It was evident that the Church was “*held in high esteem in Samoan society*.” According to one church representative,

“*at the time of the tsunami in 2009, our previous chairman (deceased) was immediately contacted by the Minister for Health for comfort. The Chairman went to the main hospital and was there during the entire day comforting the health staff and the nation at large. His prayers and speeches were broadcasted live across the nation throughout the day.*”

International and local humanitarian organizations recognise the benefits of working with churches as a powerful social agent, to advance disaster risk reduction and climate change adaptation in PICs [53, 54]. An example of the role of the church community was given by a Samoa respondent who indicated that one church in particular was the first to be on the ground when the tsunami hit. This was possible because they had all the necessary resources in terms of relief supplies and funds. They were able to immediately mobilise land and air transportation to distribute relief items, even before the Red Cross and Government representatives came in to do their assessments. They were also reported to able to provide shelter to affected communities as well as address psychosocial needs. These comments were corroborated by other Samoan respondents.

The importance of faith to build resilience was summed up by one health respondent:

*“Faith based systems and their interventions were already entrenched in the social system so people were able to turn to these during the tsunami. This is why people were more resilient than expected; they were able to recover quickly because of these social groups.” *

However, the role of the church in providing psychosocial and mental health support was also viewed as limited by some respondents. One regional representative in Fiji indicated that “*I do not think the churches have the skills or the capacity to do that….. and the churches will only speak to the Christian population. So the other people are left out-the non-Christians.”* Other respondents indicated a need to have a system in place to identify persons who are isolated from these support structures such as ethnic minorities, disabled, and those who cannot go to or are not members of a local church. The extent to which aid is exclusively provided to Church members was not determined by this study, but there was no indication that this was common practice in all case study countries. Neither was it determined to what degree Church counselling was effective. However, regardless of its impact and effectiveness, Pacific Islanders see faith-based interventions as part of their lives and culturally appropriate. It is against this background that Taylor (page 177, 2003) recommended that the ‘‘*power of religious belief and of social justice in the process of healing*” is a concept that should be carefully considered by overseas responders when working in the Pacific. [55]. This study therefore recommends that further research be conducted and that opportunities for knowledge sharing be encouraged, in order to better understand the effectiveness and importance of faith-based interventions.

## 6. Traditional Governance and Leadership

Strong traditional local governance structures are common across Pacific Island Countries, examples of which include formal Women's Committees and Council of Chiefs [53, 56, 57]. The integration of political and traditional governance has been credited for the relative stability of governance, especially in Polynesian countries [57]. Traditional governance structures are an active part of village life and play a significant role in disaster management and climate change adaptation [56, 58]. In some cases, like Samoa, their links to the national government are formalised through hierarchical traditional governance structure [56]. Understanding and incorporating the roles and essential functions of these hierarchical structures in disaster risk reduction and climate change adaptation programs can be an essential asset [53].

In Vanuatu, for example, it was reported that communities look to the Council of Chiefs for instruction and leadership in times of disasters. A health sector respondent reported that “*they *[*Chiefs*]* use a sea shell to indicate a meeting or to prepare for a Cyclone or disaster. They also ring a bell–a different type of ringing represents different type of warnings.*” Village Chiefs, who also serve as chairmen of the Area Councils (a form of local level of governance in Vanuatu), use their traditional networks in communities to share information in times of disasters. Similarly in Samoa, the Village Council is actively involved in the enforcement of adaptation activities at the community level and integration of disaster risk reduction and climate change adaptation. Evidence of this has been described through the Ministry of Women Community and Social Development's (MWCSD)Aiga ma Nuu Manuia Programme (Family Wellbeing Programme), which has gained recognition in Samoa [59]. Village Leaders reportedly ensure that “*the bell is rung to warn of impending danger such as a tsunami*.” The Women's Committees at village level are also key institutions for communities both during and after disasters. A government official in Samoa reported that

“*In times of disasters, The MWCSD mobilised these agents (village leaders and women's committees) at the local levels to ensure relief reaches the appropriate persons/household. They also feed into the national government emergency operations centre. The Women's groups help out at the disaster shelters cooking, feeding, counting wards*.”

Coordination for disaster preparedness at the village level in Samoa was reported to involve regular meetings with schools, churches, and the community to ensure that everyone is involved during disaster response. The role of traditional leadership and the active involvement of women's groups in local governance cannot be underestimated or ignored. Their involvement in the planning and implementation of disaster management and climate change adaptation programs will ensure that local knowledge and culturally appropriate approaches are applied and that there is community buy-in and support for adaptation activities.

## 7. Family and Community Involvement

The role of strong family ties and support systems as a strategy for coping with disasters was also identified. Respondents indicated that a strong extended family system was critical for support in times of disaster. In Fiji a government official noted that

“*people are strong because of the support from the family members; so when we are here in Suva and disaster strikes somewhere else, we know exactly where our relatives are. So then we can mobilise the families here to do something and then we take it to them there. Even though the Government is going to everyone, we go and see our individual families to encourage and support them*.”

A NGO representative in Vanuatu summed it up as such; “*I'm surprised at the natural resilience-they *[*communities*]* can come together at the spur of the moment when they need to.*”

Another example of the community coming together was described by respondents in Samoa. The Samoa Volunteer Emergency Response Team (VERT) and Water Safety Project (WSP) set up to strengthen emergency response capacity of the Samoa Fire and Emergency Services Authority (SFESA), the first responder to disasters and emergencies that requires immediate evacuation, search and rescue, first aid, and medical evacuation [60]. While in its early, capacity-building stages, the VERT-WSP programme aims to minimise dependence on permanent staff required to man base stations in the event of concurrent emergencies [60]. According to one respondent *“Post tsunami, the local farmers and carpenters [who were involved in the VERT] were some of the best actors in the response, because they were used to working in rough environments and this was an asset to search and rescue operations.”*

Other social support systems include the contribution of the community to the immediate needs of the affected, a common practice in PICs. In Fiji, for example, when there is an appeal for disaster relief, people donate whatever they have “*school children sometimes donate clothing and one dollar each*.” This allows the government to focus on “*funds for building the homes and supplying of building materials*.” Another example was given from Samoa, where the fact that some families own both on the coastal lands and inland plantations, facilitating moving of people from hazard zones on the coast to safer areas inland.

## 8. Agriculture and Food Security

Pacific communities have traditionally faced food security issues arising from extended dry periods associated with El Niño/La Niña-Southern Oscillation (ENSO) events or from the destruction of agricultural produce by cyclones and floods and over time have developed ways to preserve food to cope with such situations [61, 62] Climate change may bring about an exacerbation of such extremes in the Pacific [7]. Several respondents alluded to the fact that there was a need to return to *“traditional ways of preserving food to withstand during times of crisis” *with the added threats climate change may bring. However, one NGO respondent went on to say that although documentation of traditional agricultural practices is limited, “*it has been passed down from generations [so] they know what to do*.”

There has been a call from the Climate Change and Development Community (CCDC) to renew traditional knowledge of agricultural and farming practices. In some instances, they suggested that an ideal approach could be an “integrated farming system approach” that incorporates the planting of fruit trees with root crops [32]. Examples of an integrated farming approach were cited in Samoa where it was reported that current efforts by local government organizations and NGOs are “*seeking to include traditional agriculture and horticulture for food security and livelihoods through economic programs*.” These include how to plant, what are the best sites, organic methods, and how to cook the produce and the importance of traditional ways of growing. For example, one NGO respondent suggested that “*having a backup plantation for cyclone period by growing taro patches, since bananas and breadfruit trees fall down*.” Existing documentation of other traditional agricultural disaster-preparation techniques come from the Pacific Climate Change and Development Community such as cutting the stems of ready to harvest cassava plants to save them from damage during a cyclone; burying the harvest of taros, so it lasts for up to six months; or preserving breadfruit and pandanus by making them into a paste in Kiribati [32]. Also, “*in the old days,*” after a cyclone Samoan communities would salvage ready to harvest root-crop tubers, filled with valuable starch, which were then preserved in underground fermentation rooms [32].

One respondent in Fiji indicated that “*knowing that climate change will bring about risks to food we have to do things to suit us and food preservation is important.*” To this end, it was reported that “*a school teacher is preparing documents for the food security.*” However, the respondent went on to say that there was a need for funding to produce the documents, but there was limited funding for the food security program.

Another approach suggested by some respondents was to introduce less common foods and reintroduce some forgotten foods into diets. According to one respondent, “*Cassava was traditionally perceived as pig food, but efforts are being made to re-introduce it in the Samoan diet*.” In Fiji, one respondent indicated that there was a risk for overfishing, and suggested alternatives. Traditionally, tuna and Skipjack are not eaten in Fiji [63], but with changing behaviour and adapting to the overfished resources, these species may become a viable alternative in Fijian diets.

Using organic agricultural practices is one way to preserve the produce. Both Fijian and Samoa respondents mentioned that the governments were supporting the growth of pesticide free and chemical free crops. One Samoan respondent indicated that while the international market wants large fruits, those that are found in the local markets are small and pesticides-free. Another regional respondent concurred; countries with strong tourism sectors such as Fiji, and increasingly Samoa and Cook Islands, have major markets for organically grown vegetables.

However, some harmful practices were identified as compounding the negative impacts of climate change. These include the use of dynamite for fishing, use of burning to clear vegetation for planting increasing the risk of soil erosion, landslides, and flooding. Similar sentiments were expressed by members of the CCDC, citing examples of maladaptation [32]. Examples of how misunderstanding of local traditional systems and practices can undermine community resilience and adaptation in times of disaster was also described. For instance, in Samoa according to one government official, after the tsunami in 2009 some NGOs were offering ‘‘cash-for-work.” When the local authorities asked for the community's assistance later, community members requested payment. The respondent found this very unusual as previously community spirit was the default way of response. The respondent perceived this as affecting the resilience and mindset of villagers who were accustomed to working to build their own community. Furthermore, offering cash-for-work is not sustainable by the local authorities and so may have affected the adaptation capacity of the community. This is one example of how understanding the local value system is useful and how misunderstanding of such value systems may be negatively impacted by inappropriate external influences.

## 9. Limitations and Further Research

As a qualitative methodology was used, this study cannot report on the efficacy of the traditional coping strategies described. As such, these findings must be interpreted in that context. However, the rigorous methodology ensured inclusivity of a very large sample size of various sectors and stakeholders from the region, supported by literature and peer-review by subject experts from the region; providing triangulation of the broad thematic areas. Moreover the representation of Pacific Islanders on the PRG who played a critical role in informing and guiding the interpretation and analysis of research outcomes provide further validation of the findings.

Exploring a mix of countries allowed a more regional view of how disaster response plays out in different settings which proved a strength of the study. However, in terms of traditional coping strategies this broad viewpoint was also a limitation. Pacific Islands are unique in their culture and traditions, and as noted by Nunn (2009), the interpretation of the climate change concept is influenced by local culture and religion. As such traditional coping strategies that work in one country may not necessarily work the same way in another country, even across countries within the same region. As this study focussed on a regional overview, the traditional mechanisms described may be particular for the respondents' country; however, the broad thematic areas were common across all countries. Specific traditional practices for the complex and varying cultures of the Pacific would need to be explored on a very local level. Therefore further research into some of these strategies and their effectiveness in building community resilience throughout the disaster continuum is recommended.

## 10. Conclusions

This paper has provided examples of ways in which traditional practices and coping strategies for disaster response are drawn upon in the Pacific. The use of stakeholder interviews with key members of disaster response organizations in Australia and the Pacific allowed specific insight into the ways in which Pacific islanders apply traditional knowledge and coping strategies to prepare for and respond to disasters. The respondents in this study provided many examples of traditional coping strategies being used to address climate change impacts. The most important themes that emerged include recognition of traditional coping strategies; faith and religious beliefs; traditional governance and leadership; family and community involvement; and agriculture and food security. Acknowledging and preserving these useful traditional coping strategies can contribute significantly in helping the donor and NGO community in integrating them into culturally relevant programming. Furthermore they can also provide important impetus for Pacific Island governments to embrace these approaches in their disaster preparedness and response processes. While evidence of effectiveness is needed, traditional practices are still important mechanisms that are naturally integrated into the lives of Pacific Islanders as described by respondents to this study. Therefore it is important that traditional ways of operating and supporting local communities are understood by external organizations seeking to offer support and provide humanitarian response in Pacific Island Countries. Understanding the historically embedded social support systems that encourage self-help and working together is likely to enhance the outcomes of donor programs and development initiatives.

## Figures and Tables

**Table 1:** Summary of traditional coping strategies to disasters and climate changes.

Key areas	Summary of traditional knowledge and coping strategies for response to disasters and climate change in Pacific Island Countries
Recognition of traditional coping strategies	(i) Secretariat of the Pacific Community's (SPC) list of crops from the climate ready collection. (ii) Glossaries of traditional climate change terms in some Pacific countries. (iii) Pacific Red Cross societies Toolkit to assess natural disasters vulnerability and response capacity.

Faith and Religious beliefs	(i) Faith-based systems and their interventions are entrenched in the social system and can build resilience. (ii) Recognition of churches, their role in community life, including use as emergency centres or disaster shelters; provision of postdisaster counselling. (iii) Churches may be a source of resources, volunteers, welfare programs for the poor, and needy including non-members.

Traditional governance and leadership	(i) Indication of some integration of political and traditional governance systems for disaster management. (ii) Active involvement of traditional governance structures in village life, disaster management, and climate change adaptation. (iii) Use of traditional leadership networks to share information and communicate in times of disasters. (iv) Involvement of schools, churches, and the community in disaster preparedness and response.

Family and Community Involvement	(i) Extended family system and kinship ties provide a critical support structures in times of disaster. (ii) Movement of families from high risk areas to less vulnerable areas inland during disasters. (iii) Fostering of relationships improves coordination of response teams and helps develop good coping skills.

Agriculture and food security	(i) Traditional agricultural disaster-preparation techniques to preserve seedlings and seeds. (ii) Documenting food preservation techniques and consideration of inclusion in school curricula. (iii) Integrated farming system approach that incorporates the planting of fruit trees with root crops. (iv) Introducing less common foods and reintroducing some forgotten foods from traditional diets. (v) Using organic agricultural practices- pesticide free and no chemicals.
